# Phylogenetic and Divergence Time Estimation of Muscomorpha with Low-Coverage Whole-Genome Sequencing of Syrphidae (Diptera: Brachycera)

**DOI:** 10.3390/biology15050411

**Published:** 2026-03-02

**Authors:** Chunfeng Liao, Hu Li, Zhendong Gao, Wenhui Yan, Yao Ji

**Affiliations:** Shaanxi Key Laboratory of Bio-Resources, State Key Laboratory of Biological Resources and Ecological Environment of Qinling-Bashan, Qinling-Bashan Mountains Bioresources Comprehensive Development C.I.C., School of Biological Science & Engineering, Shaanxi University of Technology, Hanzhong 723000, China; 18780718231@163.com (C.L.); soar0710@foxmail.com (Z.G.); 18829378084@163.com (W.Y.); 13109143996@163.com (Y.J.)

**Keywords:** Calyptratae, Acalyptratae, phylogenomics, divergence time estimation

## Abstract

Muscomorpha is one of the most diverse groups in Brachycera, yet its higher-level phylogenetic relationships remain controversial, primarily concerning the monophyly and placement of Syrphoidea, the position of Platypezoidea, internal relationships in Calyptratae and Acalyptratae, and the non-monophyly of Eristalinae in Syrphidae. This study utilized 81 Muscomorpha species, including 22 newly sequenced Syrphidae species, and reconstructed their phylogeny with multiple datasets and models. The results confirmed monophyly of most superfamilies except Syrphoidea, with Platypezoidea as sister to the remaining Muscomorpha. Oestroidea was sister to Muscoidea. Within Syrphidae, Syrphinae monophyly and Syrphini relationships were strongly supported, while Eristalinae was non-monophyletic. Milesiini, Eristalini, Volucellini, Brachyopini, and Rhingiini were monophyletic. Divergence time estimation using MCMCTree indicated that Muscomorpha originated in the Middle Jurassic at approximately 171.66 Mya, with Syrphoidea diverging in the Late Jurassic at 151.05 Mya, Acalyptratae in the Early Cretaceous at 117.50 Mya, Calyptratae in the Late Cretaceous at 84.66 Mya, and Syrphidae at 103.44 Mya. These findings provide a robust phylogenomic framework for Muscomorpha evolution.

## Simple Summary

Flower flies are vital pollinators and pest controllers, yet their evolutionary history remains debated. We analyzed the genetic blueprints of 81 species to clarify their family tree. Our research reveals their historical relationships and ancient origins dating back to the dinosaur era, showing that some traditional groupings must be reorganized to reflect their true ancestry. By providing a clearer map of their evolution, this study enhances our understanding of insect biodiversity. Such knowledge is crucial for protecting these beneficial insects and the ecosystems they support, ensuring continued benefits for global agriculture and the natural world.

## 1. Introduction

Muscomorpha is an infraorder of Brachycera (Diptera). Worldwide, there are approximately 100,000 recorded species of flies [1]. Muscomorpha contains a wide variety of major fly groups, such as houseflies, fruit flies and bottle flies, including most of the species in Brachycera [2]. Flies in the Muscomorpha infraorder have broad diversity, including ecologies such as predation, herbivory, and xylophagy [3], while some flies also have hematophagous or parasitic characteristics, transmitting pathogens to various vertebrates that play an important role in ecological and medical fields [4]. Muscomorpha is divided into two groups, Aschiza and Schizophora [5]. Aschiza includes two subfamilies: Platypezoidea and Syrphoidea. The monophyly of Syrphoidea is still controversial, but both morphological and mitochondrial evidence suggests that the Syrphoidea is a monophyletic group [6,7,8]. However, nuclear molecular data suggests that Pipunculidae is separated from Syrphoidea [9,10,11]. Schizophora undergoes rapid radiation lineage and is one of the most diverse groups in Dipteragroups in Diptera [12]. Schizophora contains two subjections: Acalyptratae and Calyptratae; Acalyptratae is considered paraphyletic [13]. Most taxonomic systems classify Calyptratae into Hippoboscoidea, Oestroidea and Muscoidea [14]. Although the monophyly of Calyptratae is supported, the relationship between Muscoidea and Oestroidea needs to be further explored [15].

Syrphidae, commonly known as hoverflies or flower flies, belongs to the suborder Cyclorrhapha, infraorder Aschiza, and superfamily Syrphoidea; it is one of the most abundant families in Muscomorpha, with more than 6500 species [16]. Most adult Syrphidae exhibit flower-visiting behaviors and are important pollinators in natural ecosystems and agricultural crops [17]. Syrphidae larvae include herbivorous, detritivorous, and predacious species, and they inhabit diverse habitats [18]. Several different classification systems have been established for Syrphidae based on different morphological characteristics. Hull and Riley [19] used morphological synapomorphies to propose a classification system of 12 subfamilies and 25 tribes. Thompson [20] first proposed that Microdontinae was sister to a group of all other subfamilies, a view later supported by molecular studies [21,22,23,24,25]. The most widely accepted current classification is a three-subfamily system, consisting of Syrphinae, Eristalinae, and Microdontinae [26]. Some studies combining morphology with genomic data have suggested that Pipizini should be elevated to a separate subfamily [18,25,27,28]. Most contemporary research supports the monophyly of Syrphinae, Pipizinae, and Microdontinae, while the monophyly of Eristalinae remains unresolved [12,25,29].

In recent years, next-generation sequencing (NGS) has greatly expanded the availability of genomic data in systematics, increasing the number of loci that can be used for phylogeny genomic analyses [30,31]. Through low-coverage, whole-genome sequencing, a large amount of single-copy orthologous gene data from species can be obtained [32]. Currently, phylogenetic studies based on low-coverage gene sequencing have been carried out in multiple species. Boyd et al. [33] performed whole-genome sequencing on 61 species of *Columbicola* using a dataset of 977 orthologous genes to construct the most credible phylogenetic tree within *Columbicola*. Wang et al. [34] newly sequenced and analyzed the low-coverage genome sequencing of five green lacewings, reconstructing the phylogenetic relationships of Chrysopidae by using 2213 homologous gene sequences, with single-copy orthologous genes averaging 72.49% of BUSCOs, and estimated the divergence times of major lineages based on the recovered topological structure. Allio et al. [35] identified orthologous coding genes to investigate the phylogenetic relationships and divergence times of swallowtail butterflies in the family Papilionidae. Similarly, Wu et al. [36] recovered 81 single-copy orthologous genes to reconstruct the phylogeny of Syrphidae; by employing multiple datasets and analysis strategies, they found strong support for the monophyly of Syrphinae.

In this study, we employed a low-coverage whole-genome sequencing approach to extract 3285 universal single-copy orthologs (USCOs) from Muscomorpha species. USCOs, which are highly conserved genes present in a single copy across taxa, were prioritized for their advantages in phylogenomic analyses; they minimize paralogous interference, reduce compositional bias, and provide robust, unbiased evolutionary signals, making them particularly suitable for resolving complex relationships in highly diverse groups like Muscomorpha. Aligned loci were filtered based on the number of parsimony-informative sites and degree of compositional heterogeneity, generating matrices with different levels of completeness. This represents the first application of low-coverage whole-genome data to Muscomorpha phylogenomics. Additionally, we estimated divergence times within Muscomorpha using MCMCTree, providing valuable insights into the construction of higher-level phylogenetic relationships in this taxon.

## 2. Materials and Methods

### 2.1. Taxon Sampling and Species Identification

A total of 81 species within Muscomorpha were included in this phylogenetic analysis. Among these, 22 Syrphidae species were newly sequenced in this study, while the data for the remaining 59 species were retrieved from the NCBI database (http://www.ncbi.nlm.nih.gov, accessed on 16 October 2024). Of the NCBI data, 11 species were obtained as raw sequencing reads and 48 species as genomic assemblies. A comprehensive list of all 81 species, including their classification, sequencing sources, and NCBI accession numbers, is provided in Table S1 (Supplementary Material File S1). The selection of these 81 species was guided by several strategic criteria: first, to ensure broad taxonomic coverage of major lineages within Muscomorpha, particularly superfamilies with unresolved phylogenetic relationships such as Syrphoidea, Oestroidea, and Muscoidea; furthermore, to prioritize species that could significantly fill current phylogenetic gaps in Syrphidae; and additionally, to include representative taxa from both Calyptratae and Acalyptratae, along with specific outgroups, to reliably reconstruct ancestral states.

Muscomorpha sampling includes 15 superfamilies and 17 families. Among them, the Syrphidae groups involve 13 tribes, 27 genera, and 48 species; other flies total 33 species. According to the higher-level phylogenetic hypothesis of Asilidae (Diptera:Brachycera) proposed by Dikow [37], a parsimony-informative site tree was constructed, which supported the monophyly of Asilidae. Therefore, two species of Asilidae were used as outgroups, including the genomic assemblies of *Machimus rusticus* (GCA_951509405.1) and *Philonicus albiceps* (GCA_963969385.1). Syrphidae were identified by Hu Li, Juan Li, Gang Wu and Chunfeng Liao based on Huang and Cheng [38] and Huo, Ren and Zheng [26]. Voucher specimens were deposited in Professor Li’s laboratory of the School of Biological Science & Engineering, Shaanxi University of Technology Hanzhong, China.

### 2.2. DNA Extraction and Sequencing

Syrphidae specimens were collected by sweep netting, preserved in anhydrous ethanol, and stored at −20 °C for DNA extraction. DNA was extracted primarily from the thorax and head tissues; these parts were prioritized because the thorax is rich in highly developed flight muscles with high cell density and nuclear DNA, while the exclusion of the abdomen minimizes potential contamination from gut microbiota. The selected tissues were removed and ground thoroughly, and DNA was extracted using a TIANamp Genomic DNA Kit (Tiangen, Beijing, China). Extracted DNA concentrations were determined using a NanoDrop 2000 spectrophotometer (Thermo Scientific, Waltham, MA, USA). Samples with a concentration of 20 ng/μL or higher and a volume of 50 μL were sent to Berry Genomics (Beijing, China) for sequencing. Whole-genome sequencing was performed on the Illumina NovaSeq 6000 platform (Illumina, Inc., San Diego, CA, USA), generating 150 bp paired-end data with a yield of 3–8 Gb of raw sequence data per sample.

### 2.3. Genome Assembly

Quality control and normalization of all obtained sequencing data was performed using BBTools v 38.29 (https://sourceforge.net/projects/bbmap/) (accessed 11 November 2024) with error correction using Lighter v1.1.1 [39]. Genomes were assembled using Minia3 [40] based on multiple K-mer strategies to obtain contigs. Redundant contigs were removed using Redundans v0.13c [41]. Contig extension and Gap filling were performed using BESST v2.2.8 [42] and GapCloser v1.12 [43], implemented in SOAPdenovo2 [44]. Minimap2 v2.9 [45] was used to generate input mapping files for sequence elongation, and SAMtools v1.7 [46] was used to convert the mapping files into BAM format and finally complete the genome assembly for subsequent analysis. Genome assembly information can be obtained by analyzing whole-genome de novo sequencing raw data, such as sequencing coverage, genome size, Scaffold N50, and GC content.

### 2.4. Extraction of Single-Copy Orthologues

The assessment of genome completeness was performed using BUSCO v5.4.7 [47] to identify USCOs (universal single-copy orthologous genes), which is based on the Diptera reference dataset (Diptera odb10 provided by the OrthoDB homologous gene database, creation date 5 August 2020, BUSCO number 3285, https://busco-data.ezlab.org/v5/data/lineages/, accessed on 24 March 2025). Assembled genomes with a completeness of >50% or higher were selected for subsequent analysis. Since BUSCO analysis does not report fragmented or incomplete USCO sequences, the default standard deviation σ of the average length of USCO was modified to 2σ in this study to extract more USCO sequences [48]. Finally, the assessment results of all species in Muscomorpha were integrated, and the BUSCO_extraction.sh [49] script was used to extract single-copy orthologous genes from the BUSCO analysis sets. All extracted single-copy orthologous genes were translated into amino acid sequences and for subsequent analysis [34].

### 2.5. Gene Alignment and Trimming

For sequence alignment, MAFFT v7.394 [50] was used to align each loci, using the script align_MAFFT.sh and the alignment strategy mafft-auto [32]. This strategy functions as an intelligent algorithm selection policy, allowing the software to automatically switch between optimal algorithms based on the scale and characteristics of each individual gene dataset, such as sequence number, length, and similarity, thereby ensuring high alignment quality across genes with varying evolutionary rates. Subsequently, the amino acid alignments were trimmed and polished using TrimAL v1.2 [51], via the trimming_alignment.sh script, employing the automated1 heuristic method [32]. This approach effectively balances the removal of noise (regions with chaotic arrangements or excessive gaps that could compromise phylogenetic accuracy) with the preservation of essential phylogenetic signals.

### 2.6. Molecular Marker Screening and Data Matrix Construction

Three data matrices (USCO75, USCO85, and USCO95) were generated by running the script (matrix_generation.sh), representing 75%, 85%, and 95% completeness thresholds, respectively; these thresholds represent the lowest proportion of taxa in all partitions. Based on the obtained sequence data matrices, the number of parsimony-informative sites and the detection of compositional heterogeneity were calculated using PhyKit-1.11.10 [52], using the loci_filtering_alignment-based.sh script [32], and only the loci with parsimony information sites >100 were retained in the calculation of the number of parsimony information sites. Based on the number of parsimony-informative sites, compositional heterogeneity was tested, and sequences with compositional heterogeneity <0.6 were retained. Gene trees were constructed based on these sequences. Then, the symtest strategy in IQ-TREE v2.1.3 [53] was used to filter out sequences with stable, reversible, and homogeneous characteristics. The final sequences obtained were used to construct individual gene trees, spurious homolog identification was performed on these trees using SeqKit v.2.3.0 [54], and potential non-homologous gene sequences containing false homologous genes were deleted. In order to overcome the topological inconsistency [55], this study selected advanced phylogenetic information and molecular markers with ABS (Average Bootstrap Support) > 80 and used FASConCAT-g v1.05 [56] to concatenate them. Finally, three USCO matrices (USCO75_abs80, USCO85_abs80, and USCO95_abs80) were generated for subsequent phylogenetic analyses.

### 2.7. Phylogenetic Inference

In this study, maximum likelihood (ML) was employed to reconstruct phylogenetic trees based on the different data matrices. While the optimal partition schemes and substitution models were initially estimated using ModelFinder v1.7.5 [57] within IQ-TREE, we purposefully adopted a multi-model approach to assess the robustness of our phylogenetic conclusions across varying modeling frameworks. Specifically, three distinct models representing different assumptions of amino acid substitution and rate heterogeneity were used: the homogeneous model (LG), the heterogeneous model (LG + F + H4), and the heterogeneous mixture model (EX_EHO). This strategy ensures that the resulting branching patterns and nodal supports are representative of the underlying evolutionary signal rather than artifacts of a specific statistical model.

Node supports in all ML analyses were calculated using 1000 SH-aLRT replicates [58] and 1000 UFBoot2 bootstraps [59]. Consequently, a total of nine phylogenetic trees were generated based on three matrices (USCO75_abs80, USCO85_abs80, and USCO95_abs80) and the three aforementioned models. The final phylogenetic trees were visualized and refined using iTOL (https://itol.embl.de, accessed on 19 April 2024) and Adobe Illustrator 2022 (Adobe, San Jose, CA, USA) [60].

### 2.8. Divergence Time Estimation

In this study, divergence times of major lineages within Muscomorpha were estimated using the MCMCTree module in the PAML v4.9 package [61]. The estimation was based on the maximum likelihood framework with five fossil calibration points integrated from fossil records and previous research: We set the root age with a maximum of 180 Mya based on the estimated age for Brachycera by Wiegmann, Trautwein, Winkler, Barr, Kim, Lambkin, Bertone, Cassel, Bayless and Heimberg [11] and a minimum of 128 Mya based on the estimated age for the earliest divergence of Asilidae by Dikow et al. [62]. Additional calibration points were derived from the Paleobiology Database (PBDB; https://paleobiodb.org/navigator/; accessed on 30 December 2023) and relevant taxonomic literature, including that the age of Oestroidea (minus *Mystacinobia*) is estimated to be 48.2 Mya (95% HPD: 37.1 to 66.5 Mya) [63], Ephydroidea began diversifying around 80 Mya (95% HPD: 100 to 61 Mya) [64], the oldest Syrphid fossil dated to 98.17 Mya [65], and diversification within the tribe Rhingiini is represented by *Cheilosia spheginascioides* at 33.9–38.0 Mya [36]. Following the guidelines in the software manual, the quality of MCMC runs was assessed. Convergence was evaluated using Tracer v1.7.1 [66] by examining effective sample size (ESS) values from the mcmc.txt files of two independent runs. Runs were considered converged when final ESS values exceeded 200.

## 3. Results

### 3.1. Gene Assembly

In this study, we performed genome assembly on the raw reads of Syrphidae species 3–8 G and conducted statistical analysis of sequencing depth, assembled genome size, GC content, Scaffold N50, and Scaffold L50. The average sequencing depth of Syrphidae species ranged from 10.81× (*Xylota cupripurpura*) to 43.05× (*Asarkina ericetorum*). The assembled genome sizes varied from 88 Mb (*Asarkina ericetorum*) to 582 Mb (*Volucella nigricans*). GC content ranged from 31.9% (*Epistrophe annulitarsis*) to 42.5% (*Phytomia errans*). Scaffold N50 was between 16,525 bp (*Paragus bicolor*) and 63,931 bp (*Volucella latifasciata*). Scaffold L50 ranged from 1620 bp (*Asarkina ericetorum*) to 10,832 bp (*Cheilosia ochreipila*). Detailed statistics are provided in the Supplementary Material File S1 (Table S1).

### 3.2. Identification and Analysis of Single-Copy Orthologues

**Figure 1:**
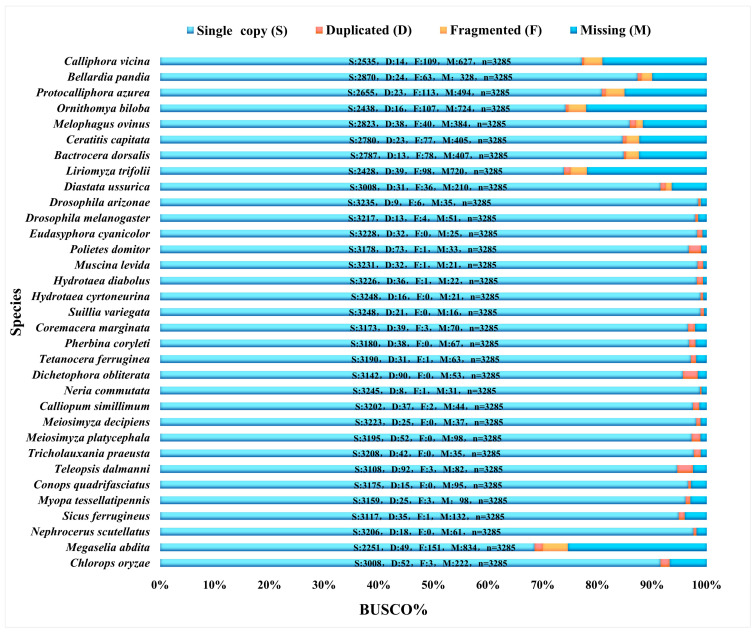
The BUSCO completeness of Muscomorpha species reanalyzed from NCBI data, with complete single copy (S, light blue), complete duplicated (D, red), fragmented (F, yellow), and missing (M, dark blue).

**Figure 2:**
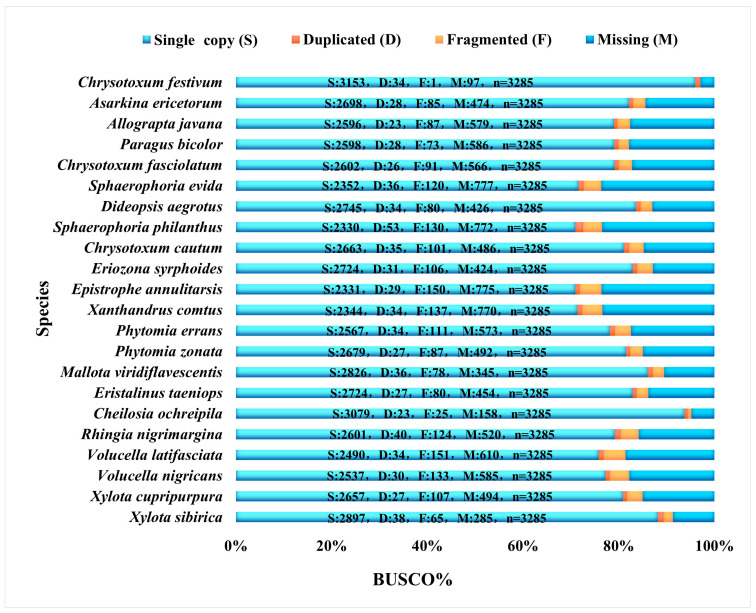
The BUSCO completeness of newly sequenced Syrphidae species, with complete single copy (S, light blue), complete duplicated (D, red), fragmented (F, yellow), and missing (M, dark blue).

**Figure 3:**
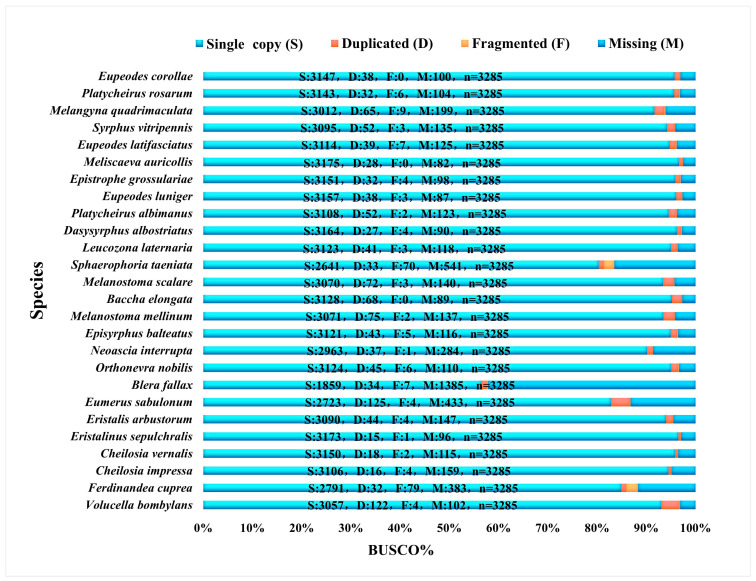
The BUSCO completeness of Syrphidae species reanalyzed from NCBI data, with complete single copy (S, light blue), complete duplicated (D, red), fragmented (F, yellow), and missing (M, dark blue).

Based on the Diptera odb10 reference dataset of Diptera provided by the OrthoDB homologous gene database (creation date 5 August 2020, BUSCO number 3285, https://busco-data.ezlab.org/v5/data/lineages/, accessed on 24 March 2025), this study concludes the BUSCO completeness assessment: the BUSCO completeness range of 83 species (Figure 1, Figure 2 and Figure 3) was from 57.6% (*Blera fallax*) to 99.5% (*Suillia variegata*). The total recovered BUSCO loci ranged from 1893 to 3269, with an average of 2971 loci obtained. Duplicated loci ranged from 0.2% to 3.8%. Duplicated sequence lengths ranged from 8 to 125. Fragmented loci ranged from 0% to 4.6%. Fragmented sequence lengths ranged from 0 to 151. Missing loci ranged from 0.5% to 42.2%. Missing lengths ranged from 16 to 1385. Detailed information is provided in the Supplementary Material File S2 (Table S2).

### 3.3. Molecular Screening

Through a series of stringent quality control and molecular filtering assessments, we obtained a high-resolution dataset for phylogenetic reconstruction. From the initial 3285 single-copy orthologous genes, 2659 loci were retained after filtering for parsimony-informative sites (>100). Subsequent assessments of relative composition variability (RCV) and symmetry (*p* < 0.05) further refined the dataset to 2579 loci. After homogeneity checks and the removal of spurious homologous sequences, 2546 loci remained. The final dataset consisted of 1831 high-quality loci after excluding genes with weak phylogenetic signals (ABS > 80). These loci provided the robust foundational data for our subsequent phylogenetic analysis.

### 3.4. Matrix Construction

In this study, three completeness matrices (USCO75, USCO85, and USCO95) were generated, representing the minimum proportion of taxa required across all partitions. Their ABS values are all above 80. Specifically, they were USCO75_abs80, USCO85_abs80, and USCO95_abs80. Among them, USCO75_abs80 contains 377,383 amino acids and 531 USCO loci; USCO85_abs80 contains 189,679 amino acids and 288 USCO loci; and USCO95_abs80 contains 29,614 amino acids and 49 USCO loci. The detailed statistics are shown in Table 1.

### 3.5. Phylogenetic Relationships Within Muscomorpha

**Figure 4:**
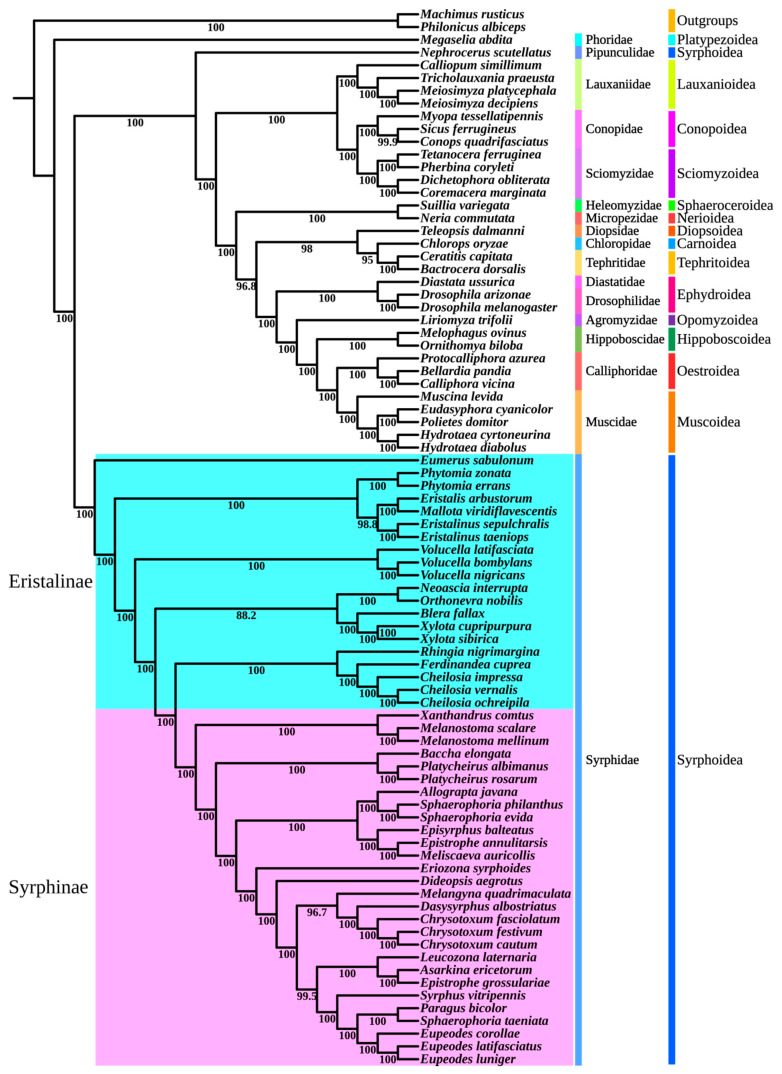
Phylogenetic tree of Muscomorpha reconstructed based on the homogeneous model (LG) using the USCO85 matrix.

In this study, new data were obtained using low-coverage whole-genome sequencing, and utilizing multiple datasets better revealed the phylogenetic relationships within Muscomorpha (Figure 4). Three tree-building strategies were applied to three data matrices; however, most nodes of the constructed phylogenetic trees remained consistent, and the higher the completeness of the matrix, the higher the support of the phylogenetic tree constructed. The final amino acid dataset comprised 81 Muscomorpha species representing 15 families, with two Asilidae species serving as outgroups. The superfamilies included in Muscomorpha are: Carnoidea, Platypezoidea, Conopoidea, Diopsoidea, Lauxanioidea, Nerioidea, Sciomyzoidea, Sphaeroceroidea, Muscoidea, Ephydroidea, Opomyzoidea, Tephritoidea, Hippoboscoidea, and Oestroidea.

The nine phylogenetic trees constructed from different data matrices and models (Supplementary Material File S3-Figure S1) provided robust support for the evolutionary relationships within Muscomorpha. All analyses recovered Platypezoidea as the sister group to the remaining Muscomorpha, consistent with previous findings [13,15,46,67]. The monophyly of Syrphoidea was not recovered; instead, Pipunculidae was consistently identified as the sister group to non-Syrphoidea Muscomorpha, supporting earlier studies [11,22,68]. The monophyly of Lauxanioidea is controversial, with some studies considering it paraphyletic, while Conopoidea and Sciomyzoidea are monophyletic sister taxa [67]. In this study, the monophyly of Lauxanioidea, Conopoidea, and Sciomyzoidea was supported, and a consistent topology was supported by the analyses of the nine trees: Lauxanioidea+ (Conopoidea + Sciomyzoidea).

The monophyly of Sphaeroceroidea, Nerioidea, Diopsoidea, and Carnoidea was supported, though the relationship between Sphaeroceroidea and Nerioidea differs across analyses, which may be due to the limited representation of these four superfamilies. In the analyses (LG USCO75, LG + F + H4 USCO75, EX_EHO USCO75, LG USCO85, LG + F + H4 USCO85, and EX_EHO USCO85), Sphaeroceroidea was sister to Nerioidea. However, in the other trees (LG USCO95, LG + F + H4 USCO95, and EX_EHO USCO95), Nerioidea was sister to Tephritoidea, with Sphaeroceroidea being sister to (Nerioidea + Tephritoidea). The monophyly of Tephritoidea is supported, but its relationships also differ between analyses. In four analyses (LG USCO85 and three USCO75 datasets), we found Diopsoidea + (Carnoidea + Tephritoidea), while other analyses (LG + F + H4 USCO85) supported Diopsoidea + (Tephritoidea + (Carnoidea + Opomyzoidea)); however, some analyses (EX_EHO USCO85) supported Tephritoidea + (Diopsoidea + (Carnoidea + Opomyzoidea)). This study supported the monophyly of Ephydroidea, consistent with previous research results [10,15,67]. The monophyly of Opomyzoidea, Hippoboscoidea, Oestroidea, and Muscoidea was supported, and these four superfamilies were shown in the analyses (LG USCO85 and three USCO75 datasets) as Opomyzoidea + (Hippoboscoidea + (Oestroidea + Muscoidea)), with all trees supporting Oestroidea and Muscoidea as sisters.

**Figure 5:**
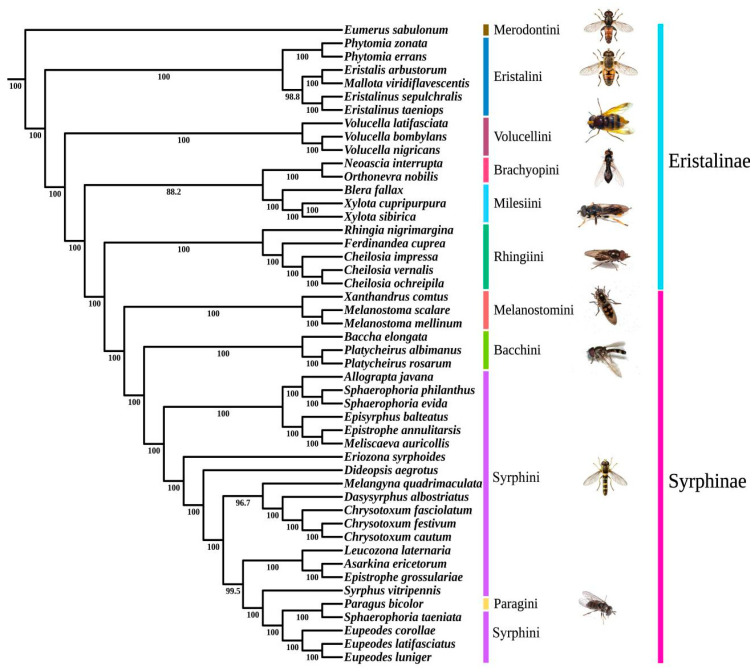
Phylogenetic tree of Syrphidae reconstructed based on the homogeneous model (LG) using the USCO85 matrix.

All analyses recovered the monophyly of Syrphidae, and the monophyly of Syrphinae was recovered, but the monophyly of Eristalinae was not recovered, consistent with previous research results [22,25,36,69]. All analyses demonstrated that Merodontini was a sister group of the other Syrphidae species, which may be due to the fact that the larvae of Merodontini are herbivorous [18,38], and the systematic relationships of Merodontini have also been proven in other scholars’ studies [21]. Additionally, the monophyly of Brachyopini, Milesiini, Eristalini, Volucellini, and Rhingiini was supported in this study, with Milesiini and Brachyopini being sister groups, while Rhingiini was identified as a sister to Syrphinae (Figure 5).

Within Syrphinae, Bacchini, Melanostomini, Paragini, and Syrphini were all monophyletic. Melanostomini was positioned as sister to (Bacchini + (Syrphini + Paragini)), consistent with recent studies [18,36,70]. The relationships among genera within Syrphini were strongly supported, including *Allograpta* as the sister to *Sphaerophoria*, *Chrysotoxum* and *Dasysyrphus* and the monophyly of Eupeodes, supported by all analyses. However, the phylogenetic positions of some genera within Syrphini varied among different trees. In the analyses (LG USCO75, LG + F + H4 USCO75, EX_EHO USCO75, LG USCO85), it was suggested that *Eupeodes* should be placed at the bottom. The analyses (LG + F + H4 USCO85, the LG USCO95, and the EX_EHO USCO95) suggest that the branch formed by *Asarkina* and *Sphaerophoria* should be placed at the bottom of the tree. The analyses (EX_EHO USCO85, LG + F + H4 USCO95) suggest placing *Chrysotoxum* at the bottom of the tree.

### 3.6. Estimation of Divergence Times

**Figure 6:**
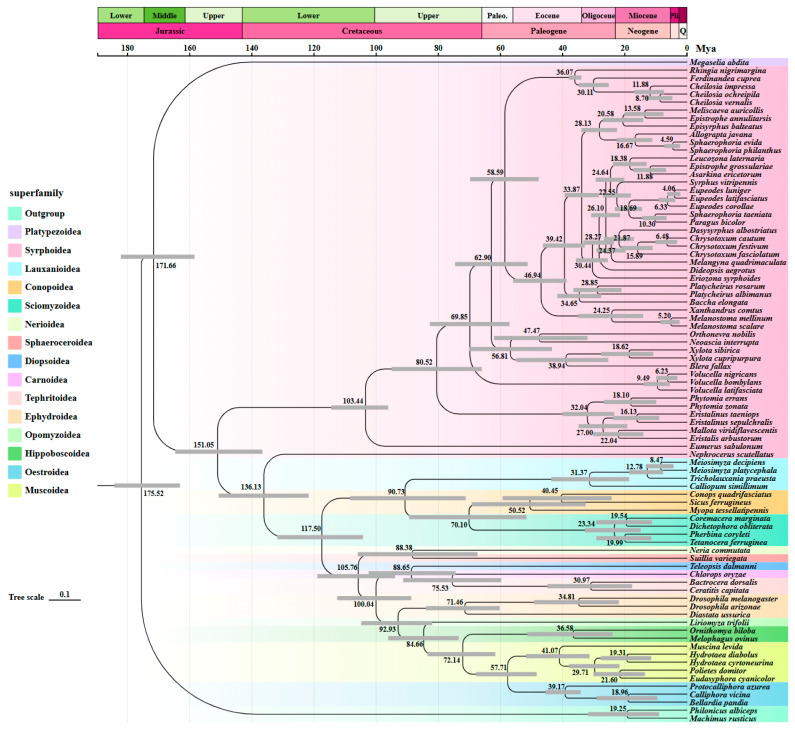
Divergence times of Muscomorpha inferred using MCMCTree. The phylogenetic tree was constructed using the LG model with the USCO85 amino acid dataset, highlighting the 95% highest posterior density of the estimated divergence times.

In this study, divergence times for Muscomorpha were inferred using MCMCTree (Figure 6), revealing the evolutionary timeline of this group from the Middle Jurassic to the Pliocene. The root age was estimated at 175.52 Mya (95% HPD: 163.11–184.21 Mya), a time interval encompassing both the Early and Middle Jurassic. This age range is highly consistent with the estimated origin of Brachycera by Wiegmann, Trautwein, Winkler, Barr, Kim, Lambkin, Bertone, Cassel, Bayless and Heimberg [11], suggesting that the early divergence of Muscomorpha as the crown group of Brachycera likely occurred during the Middle Jurassic. The lineage containing Syrphoidea diverged from the ancestors of Calyptratae and Acalyptratae approximately 151.05 Mya (95% HPD: 136.60–164.64 Mya) in the Late Jurassic, a period coinciding with a critical phase in gymnosperm evolution that provided diverse ecological niches for early Muscomorpha. The crown age of Calyptratae (including Oestroidea, Muscoidea, and Hippoboscoidea) was estimated at 84.66 Mya (95% HPD: 73.44–96.15 Mya), falling within the Late Cretaceous. The crown age of Acalyptratae was estimated at approximately 117.50 Mya (95% HPD: 104.21–131.79 Mya). This node marks the early diversification of Acalyptratae during the Early Cretaceous, laying the genetic foundation for their ecological adaptation during the subsequent angiosperm radiation. The common ancestor of Syrphidae diverged at 103.44 Mya (95% HPD: 93.90–118.95 Mya).

## 4. Discussion

In this study, the BUSCO assessment results showed that sequencing completeness of the selected species was high (57.6–99.5%), with an average of 2971 USCO sequences obtained per species. Our study demonstrates the feasibility of extracting multiple markers from low-coverage whole-genome data sequencing of Diptera [32,36].

The phylogenetic relationships of Muscomorpha were studied using single-copy orthologous genes. The monophyly of Aschiza was not recovered because the results of this study suggest separating Pipunculidae and Syrphidae in Syrphoidea, which is consistent with the results shown by some molecular data [6,7,8,10]. The monophyly of Calyptratae was confirmed, and its cluster formed almost as (Hippoboscoidea + (Muscoidea + Oestroidea)), which is consistent with the conclusions of Li, Yan and Li [15]. The monophyly of Hippoboscoidea has always been controversial; in our analysis, the monophyly of Hippoboscoidea was consistently recovered, a result that clarifies the topological inconsistencies reported in the previous literature. Earlier investigations using a 187-taxon dataset found that the position of Hippoboscoidea fluctuated between being a sister group to, or nested within, the muscoids and oestroids [71]. Compared to the clear monophyly of subfamilies in Calyptratea, the internal relationships of Acalyptratae remain unclear, and this study did not recover its monophyly. Phylogenetic trees constructed based on different models and matrices supported the division of Ephydroidea in Acalyptratae as the sister group of Calyptratea, and this result is also consistent with previous morphological and molecular evidences [9,11,67,72].

Based on traditional morphology, mitochondrial genome data, and low-coverage whole-genome methods, the monophyly of Syrphidae has been supported [9,11,22,25,36]. Although the monophyly of Eristalinae was not recovered here, the present study strongly confirms the phylogenetic relationships among genera within Eristalinae. The taxonomic status of Milesiini, however, has always been controversial [73]. Some considered Eristalini sister to Milesiini, while several studies have demonstrated the paraphyly of Milesiini [21,24,36,70]. In this study, two species of *Xylota* clustered together and formed a sister with *Blera*, and we recovered the monophyly of Milesiini; however, this may be due to the limited taxon selection.

The taxonomic status of *Platycheirus* and *Melanostoma* in Syrphinae has long been debated. Some scholars believe that *Platycheirus* and *Melanostoma* should be classified under the tribe Melanostomini [74,75], while this study would place *Platycheirus* in Bacchini and *Melanostoma* in Melanostomini, which is supported by some research [18,25,36,69]. Finally, all analyses support the sister group relationship between *Chrysotoxum* and *Dasysyrphus*, confirming Vockeroth’s [76] proposal to include *Chrysotoxum* in Syrphini.

From a paleogeographic perspective, the continuous fragmentation of Pangea during the Middle Jurassic induced significant geographic isolation, providing the evolutionary impetus for the diversification of early dipteran lineages [77]. A pivotal event in this timeline was the divergence of the Syrphoidea clade from the common ancestor of Calyptratae and Acalyptratae during the Late Jurassic; during this period, the abundance of gymnosperm secretions, such as pollination drops, likely served as the primary liquid nutrient source for early Muscomorpha, thereby driving the early specialization of their mouthpart structures [78].

The early diversification of Acalyptratae during the Early Cretaceous highly coincided with the initial global radiation of angiosperms; this temporal alignment suggests that the Acalyptratae lineage had established its fundamental genetic foundation prior to the ecological dominance of flowering plants. Such an early establishment likely provided a first-mover advantage, enabling the group to occupy diverse anthophilous (flower visiting) and phytophagous niches during the subsequent angiosperm explosion. In contrast, the establishment of Calyptratae during the Late Cretaceous may have been primarily driven by the expansion of saprophagous and parasitic niches resulting from the diversification of vertebrates, particularly the ancestors of modern mammals and birds [79].

This Cretaceous origin, Cenozoic explosion evolutionary pattern of Syrphidae reveals a robust resilience in the face of global catastrophic events; the ecological niche vacancies that emerged following the K-Pg boundary, including the turnover of pollination mediators and shifts in predatory pressures, significantly catalyzed the predatory radiation within the Syrphidae lineage [11]. This process not only spurred dramatic morphological diversification but also facilitated the transition of the group into its modern ecological role as the second most critical pollinator in contemporary ecosystems, surpassed only by bees [80].

## 5. Conclusions

This study utilizes a low-coverage whole-genome approach to clarify the phylogenetic relationships among Muscomorpha superfamilies and higher-level groups within Syrphidae. Our results confirm several previous morphological and molecular hypotheses while providing new insights into lineage relationships; specifically, our phylogenomic data reveal that the tribe Syrphini is polyphyletic, a finding that challenges traditional taxonomic arrangements.

However, taxonomic sampling in this study remains limited. Certain key groups, such as Nemestrinoidea within Muscomorpha, as well as members of Microdontinae and Pipizinae within Syrphidae, were not included. Consequently, the comprehensive phylogenetic framework of Muscomorpha and the precise taxonomic status of Microdontinae and Pipizinae remain to be fully resolved in future studies with broader taxon sampling.

The MCMCTree results indicate that in the evolutionary timeline of Muscomorpha from the Middle Jurassic (171.66 Mya) through the Miocene, the Syrphoidea lineage diverged during the Late Jurassic (151.05 Mya). Within Schizophora, Acalyptratae began diversifying early in the Lower Cretaceous (117.50 Mya), securing a first-mover advantage prior to the major radiation of angiosperms. In contrast, Calyptratae emerged later during the Late Cretaceous (84.66 Mya). Furthermore, the radiation of Syrphidae, estimated at 103.44 Mya in this study, potentially suggests a diversification phase associated with the Cretaceous Terrestrial Revolution (KTR). This timing coincides with the rapid rise of angiosperms, which likely provided vast new floral ecological niches for early syrphid lineages. In the future, multi-omics techniques such as third-generation sequencing and transcriptomics data can be used to comprehensively explain and study the specific characteristics of Muscomorpha. Based on a systematic phylogenetic framework constructed from multiple molecular markers, efforts can be made to combine important morphological features of various populations to reconstruct the ancestral state of Muscomorpha and explore the evolutionary relationships of their morphological traits.

## Figures and Tables

**Table 1:** Summary of USCO amino acid used in this study.

Matrix	Average Taxa per Locus (%)	Average Missing Taxa per Locus (%)	Number of Loci	Number of Sites	Missing Sites (%)	Average Locus Length
USCO75	85.9	14.13	531	377,383	14.69	710.7
USCO85	91.1	8.85	288	189,679	9.12	658.6
USCO95	96.2	3.76	49	29,614	4.01	604.4

## Data Availability

The NCBI Reference Sequence is listed in Supplementary Material File S1-Table S1.

## Supplementary Materials

The following supporting information can be downloaded at https://www.mdpi.com/article/10.3390/biology15050411/s1, File S1-Table S1 provide taxa sampling and basic statistics for genome assemblies and USCO extraction. File S2-Table S2 provide the BUSCO completeness for species of Muscomorpha and Syrphidae. File S3-Figure S1 provide the phylogenetic trees that were generated using distinct phylogenetic inference models tailored to different datasets.
