# Supplementary material for: Phylogenetic and Divergence Time Estimation of Muscomorpha with Low-Coverage Whole-Genome Sequencing of Syrphidae (Diptera: Brachycera)
Source: Biology (Basel). 2026 Mar 2;15(5):411. doi: 10.3390/biology15050411 (PMC12984426; doi:10.3390/biology15050411)
Supplement: Supplementary file 1 [file biology-15-00411-s001.zip › File S3-Figure S1.pdf]

The phylogenetic trees presented in File S3-Figure S1 are generated using distinct phylogenetic inference models tailored to different datasets.

Figure	Matrix	Phylogenetic inference model
Fig S1a	Matrix75	heterogeneous mixture model
Fig S1b	Matrix75	heterogeneous model
Fig S1c	Matrix75	homogeneous model
Fig S1d	Matrix85	heterogeneous mixture model
Fig S1e	Matrix85	heterogeneous model
Fig S1f	Matrix95	heterogeneous mixture model
Fig S1g	Matrix95	heterogeneous model
Fig S1h	Matrix95	homogeneous model

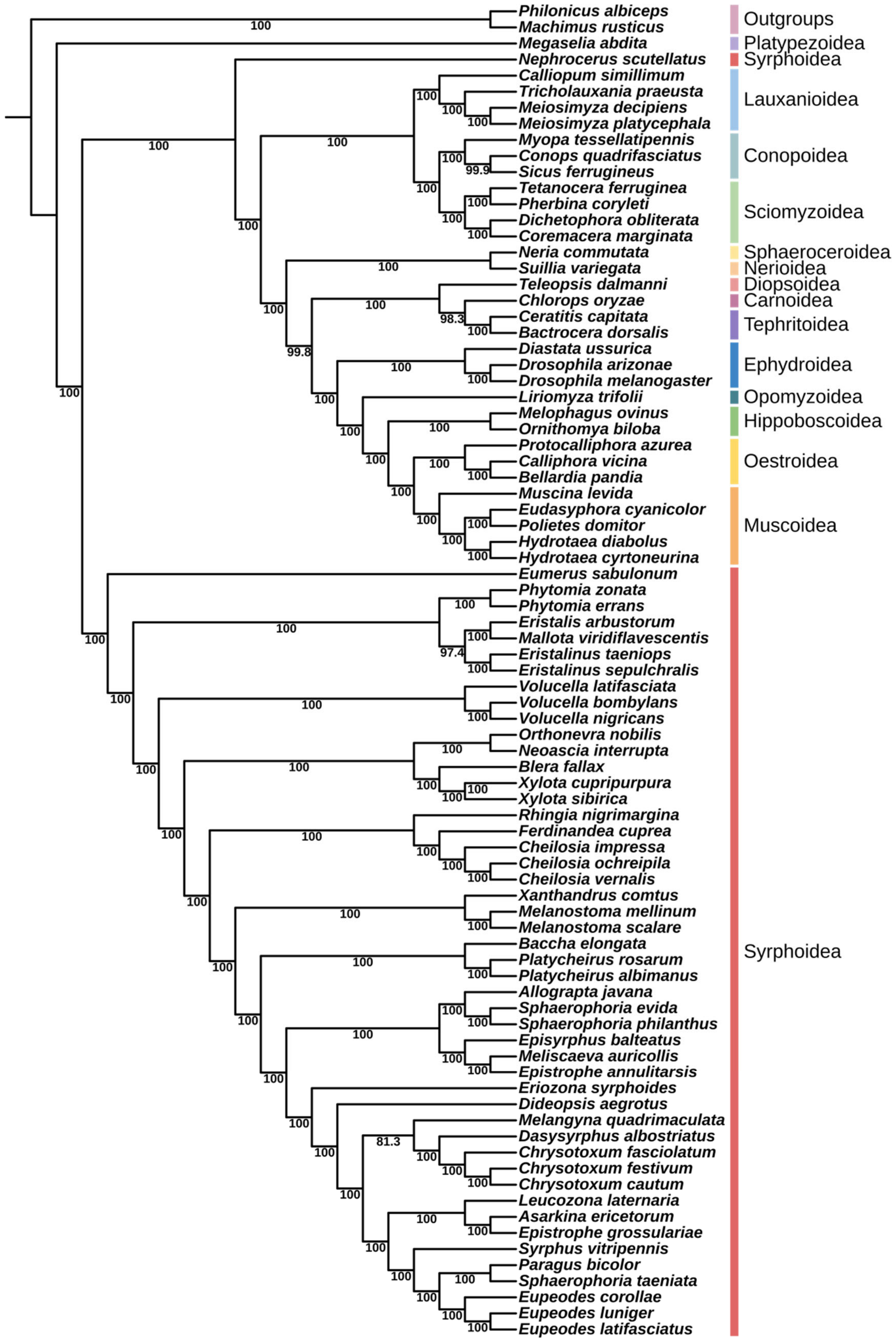

Fig S1a. Phylogeny of Muscomorpha inferred from matrix USCO75 using the heterogeneous mixture model

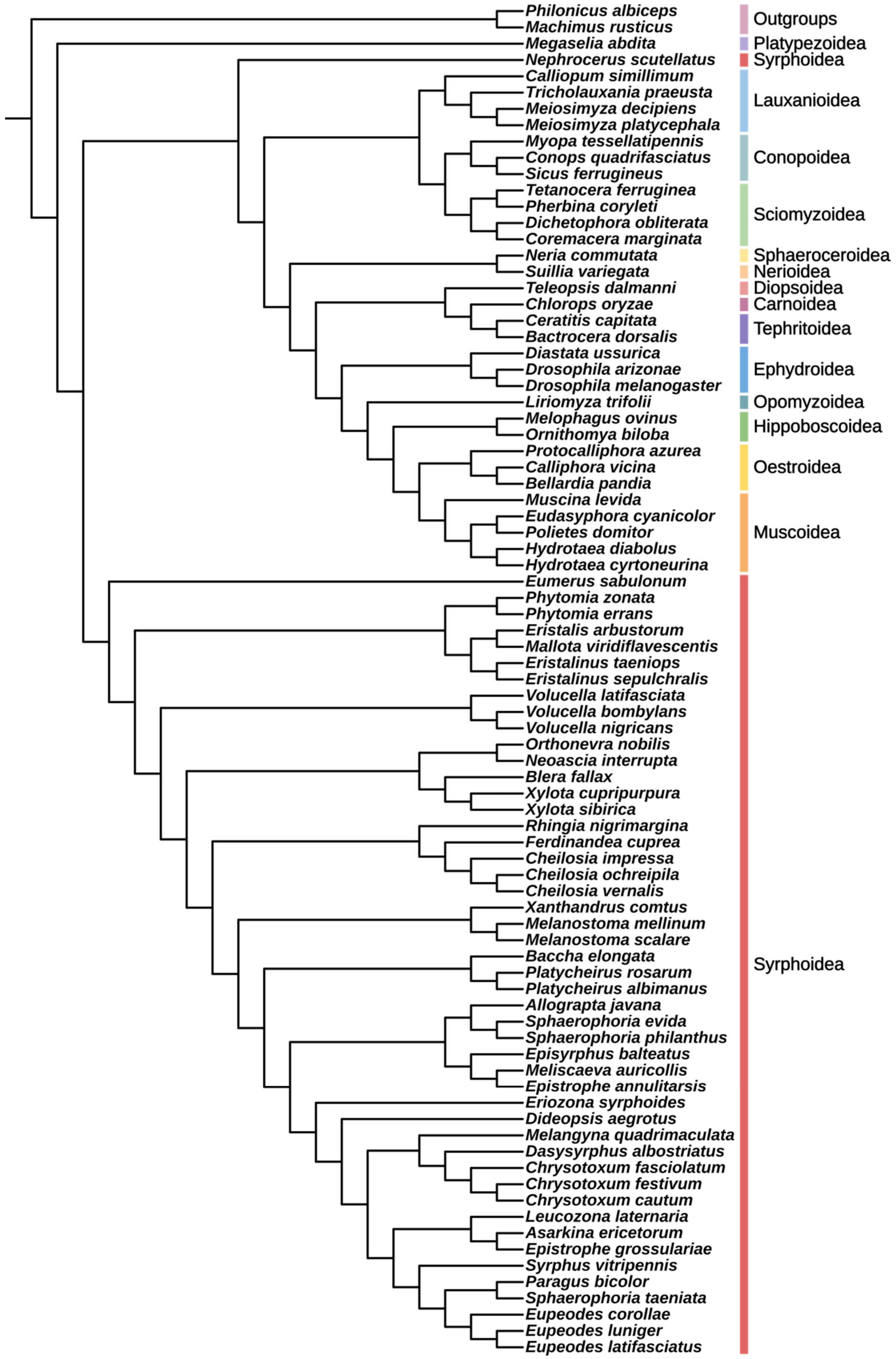

Fig S1b. Phylogeny of Muscomorpha inferred from matrix USCO75 using the heterogeneous model

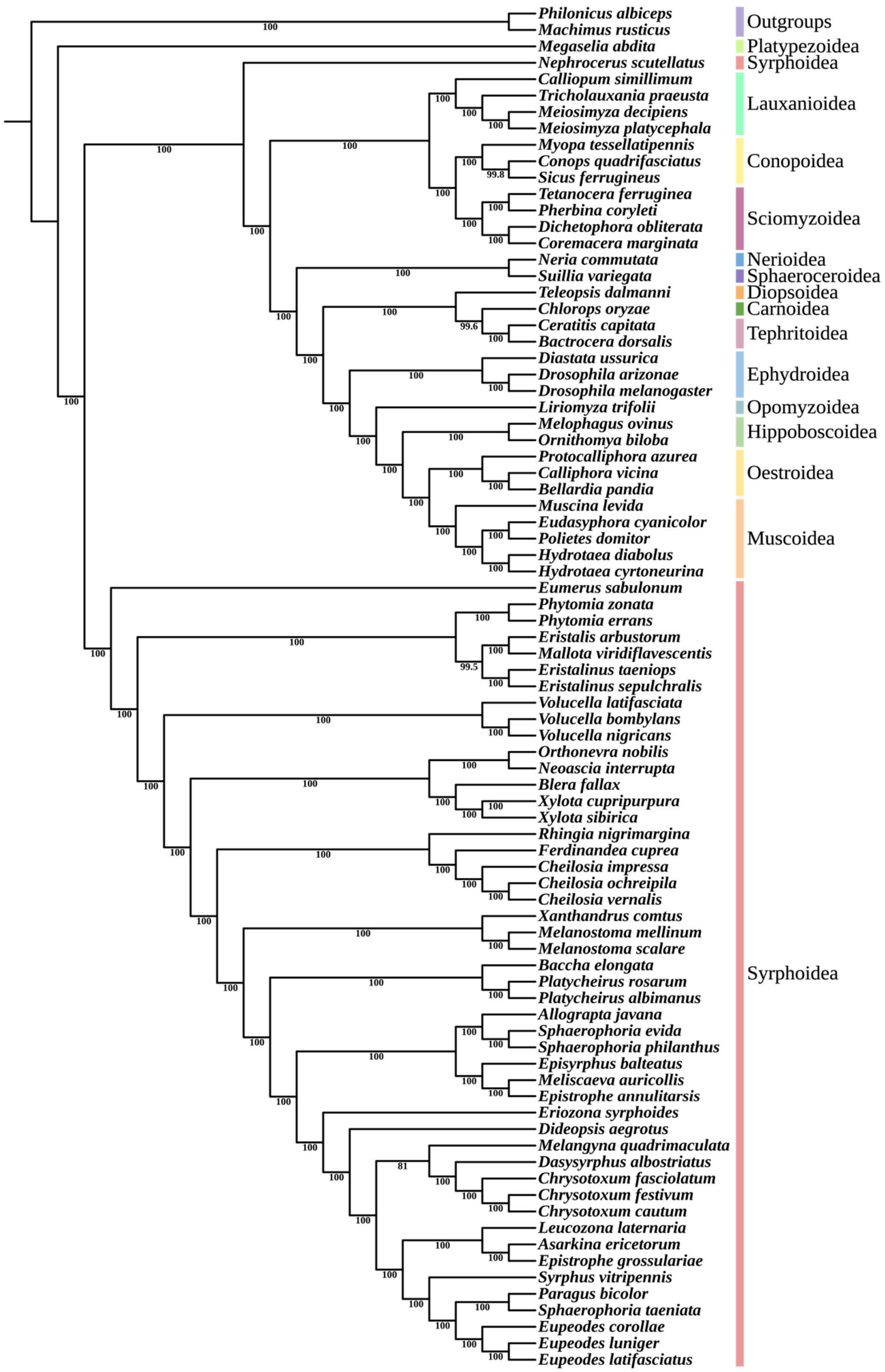

Fig S1c. Phylogeny of Muscomorpha inferred from matrix USCO75 using the homogeneous model

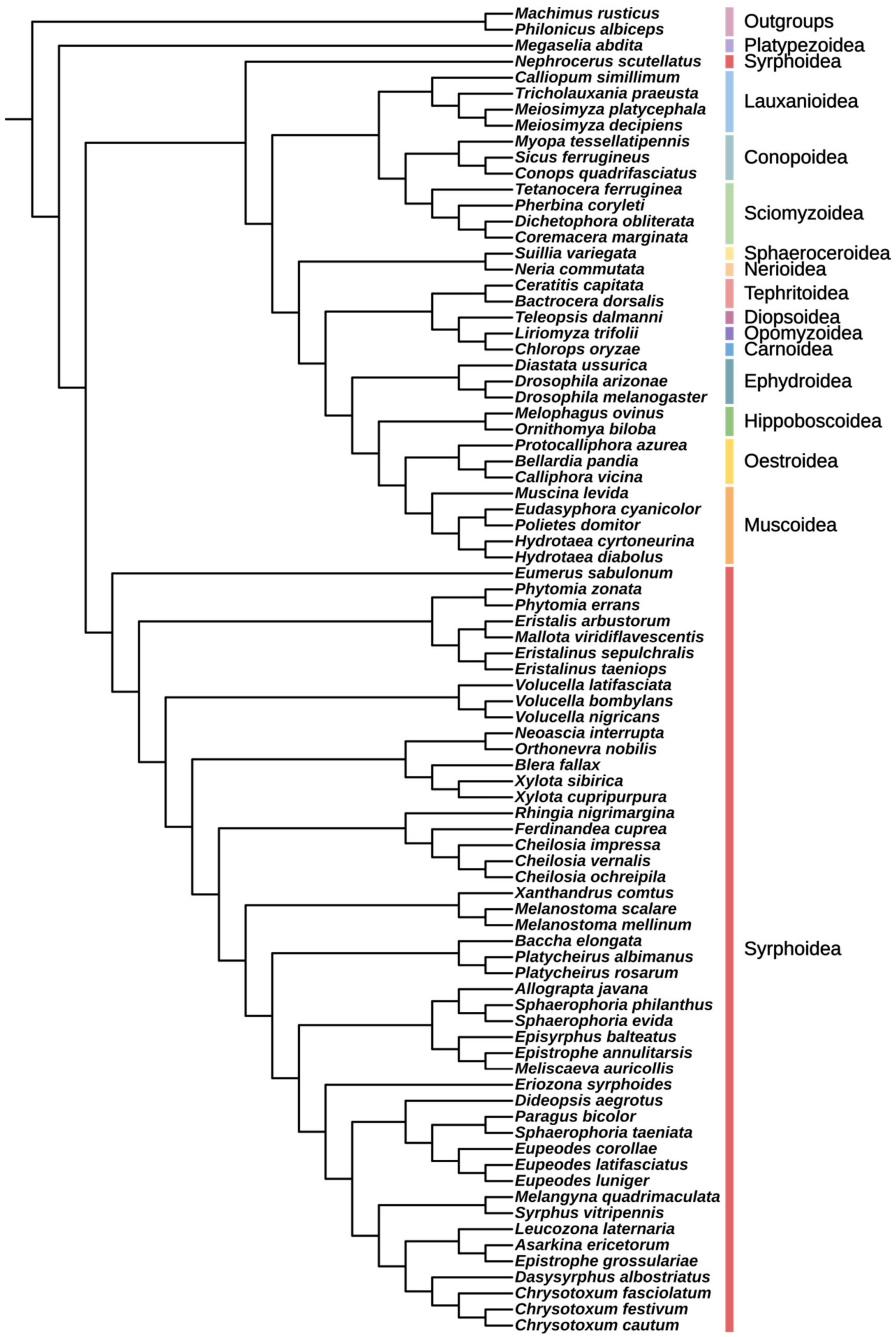

Fig S1d. Phylogeny of Muscomorpha inferred from matrix USCO85 using the heterogeneous mixture model

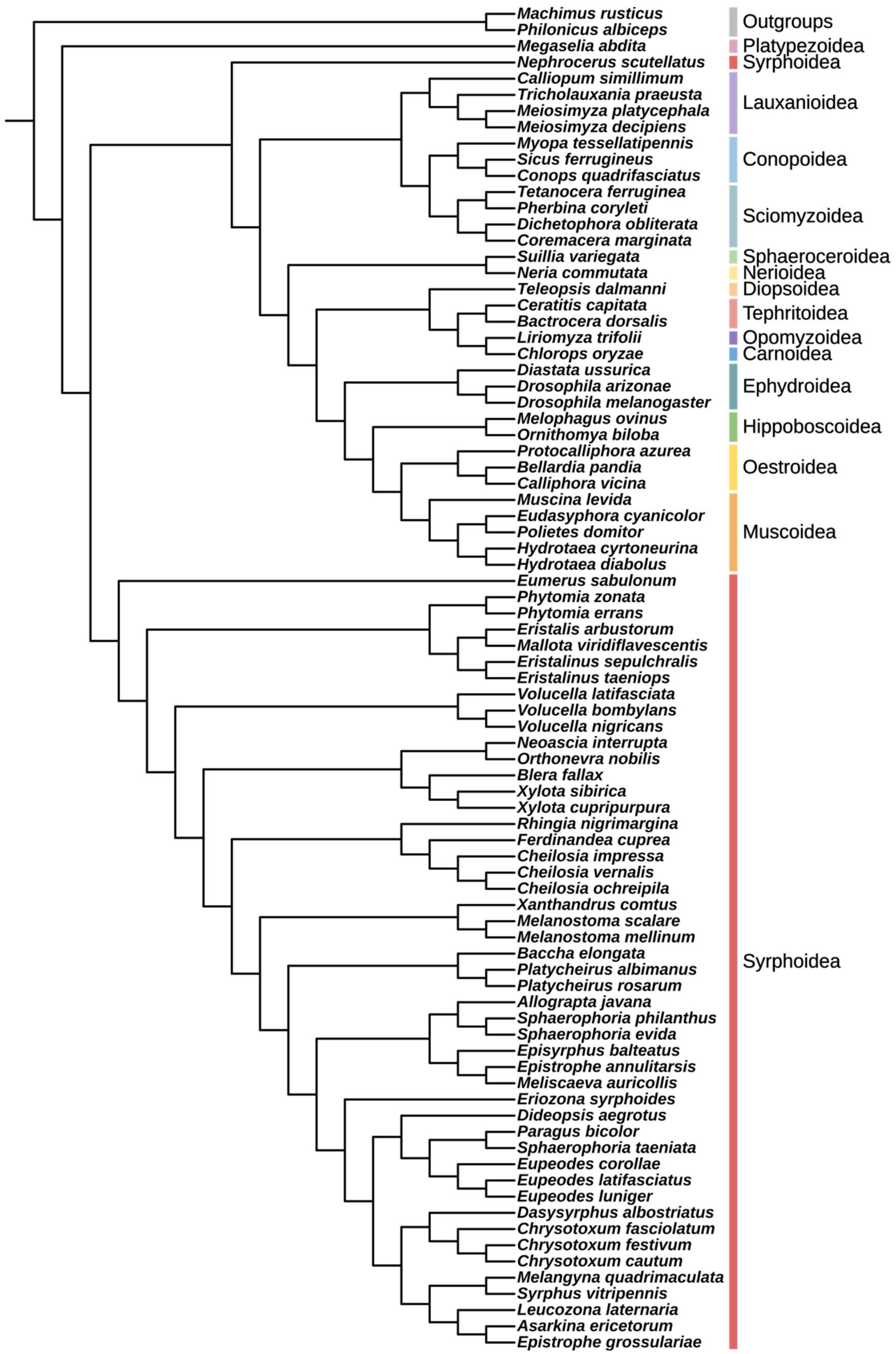

Fig S1e. Phylogeny of Muscomorpha inferred from matrix USCO85 using the heterogeneous model

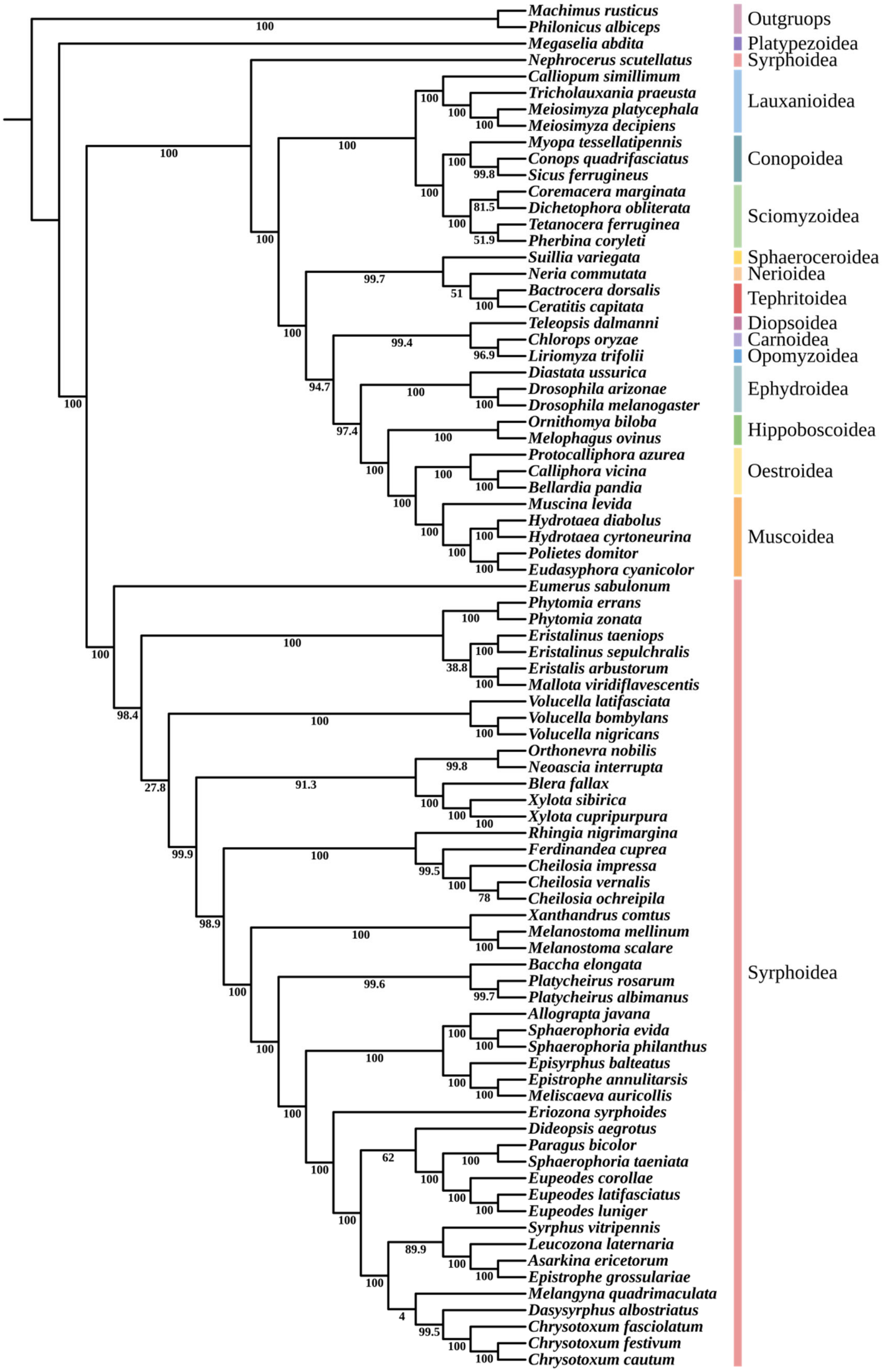

Fig S1f. Phylogeny of Muscomorpha inferred from matrix USCO95 using the heterogeneous mixture model

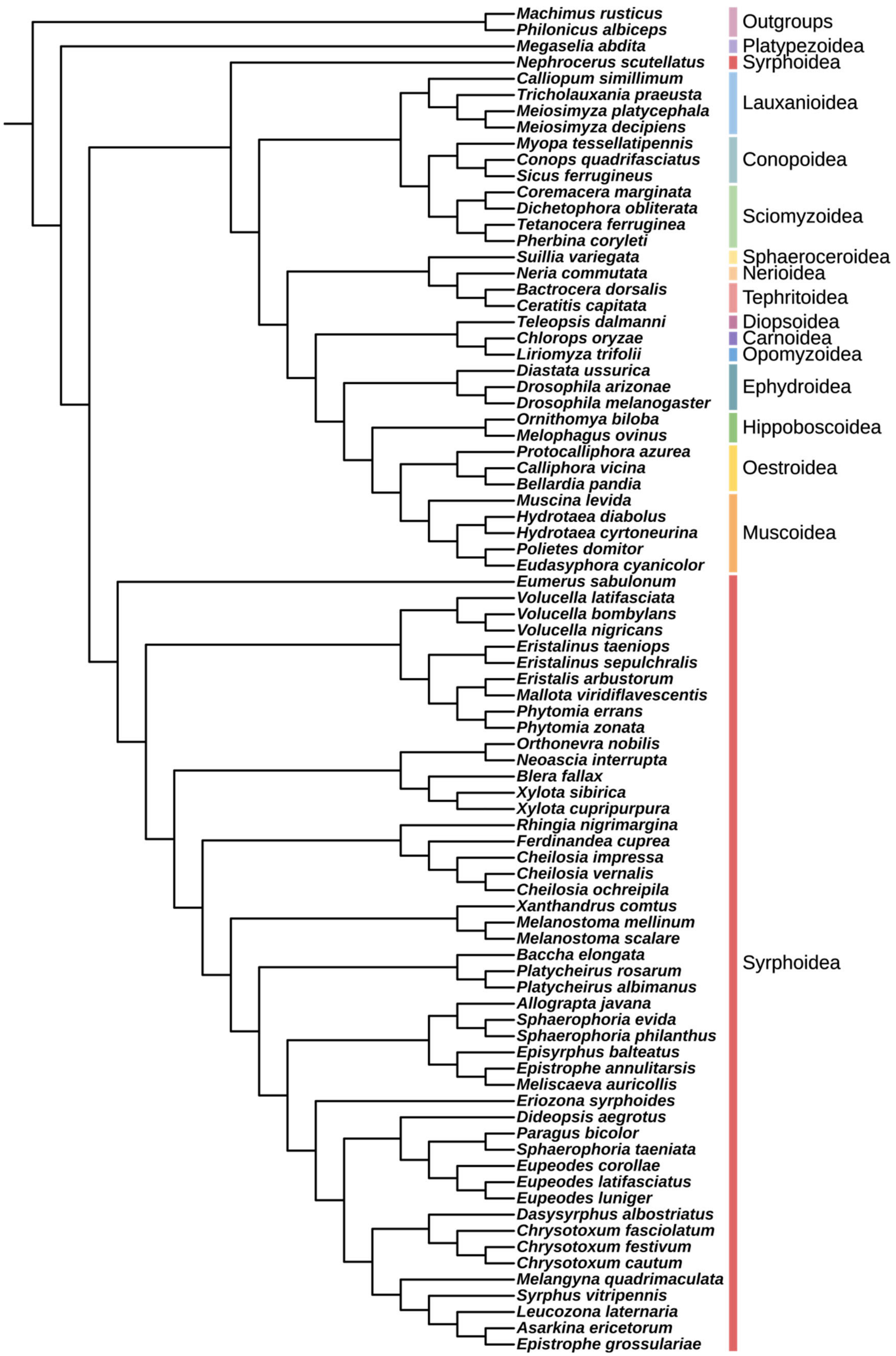

Fig S1g. Phylogeny of Muscomorpha inferred from matrix USCO95 using heterogeneous model

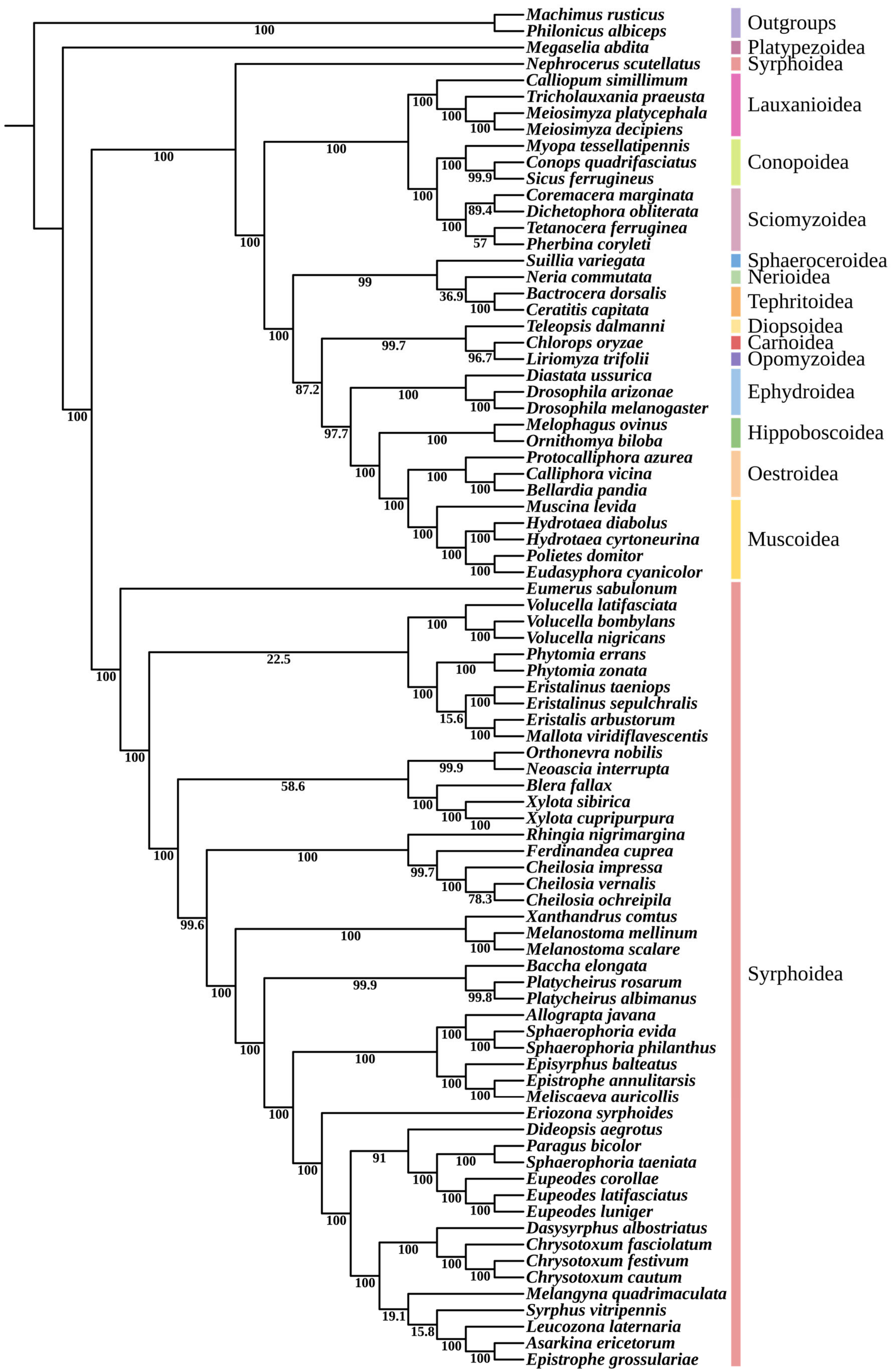

Fig S1h. Phylogeny of Muscomorpha inferred from matrix USCO95 using the homogeneous model
